# ctDNA guided adjuvant chemotherapy versus standard of care adjuvant chemotherapy after curative surgery in patients with high risk stage II or stage III colorectal cancer: a multi-centre, prospective, randomised control trial (TRACC Part C)

**DOI:** 10.1186/s12885-023-10699-4

**Published:** 2023-03-20

**Authors:** Susanna Slater, Annette Bryant, Hsiang-Chi Chen, Ruwaida Begum, Isma Rana, Maria Aresu, Clare Peckitt, Oleg Zhitkov, Retchel Lazaro-Alcausi, Victoria Borja, Rachel Powell, David Lowery, Michael Hubank, Thereasa Rich, Gayathri Anandappa, Ian Chau, Naureen Starling, David Cunningham

**Affiliations:** 1 Royal Marsden Hospital NHS Foundation Trust, London, UK; 2 Biomedical Research Centre at the Royal Marsden and the Institute of Cancer Research, London, UK; 3 Centre for Molecular Pathology at the The Royal Marsden Hospital and Institute of Cancer Research, Sutton, UK; 4 Guardant Health, INC, Redwood City, CA USA

**Keywords:** Colorectal cancer, ctDNA, Adjuvant chemotherapy, Randomised, Disease free survival

## Abstract

**Background:**

Circulating tumour DNA (ctDNA) to detect minimal residual disease (MRD) is emerging as a biomarker to predict recurrence in patients with curatively treated early stage colorectal cancer (CRC). ctDNA risk stratifies patients to guide adjuvant treatment decisions. We are conducting the UK’s first multi-centre, prospective, randomised study to determine whether a de-escalation strategy using ctDNA to guide adjuvant chemotherapy (ACT) decisions is non-inferior to standard of care (SOC) chemotherapy, as measured by 3-year disease free survival (DFS) in patients with resected CRC with no evidence of MRD (ctDNA negative post-operatively). In doing so we may be able to spare patients unnecessary chemotherapy and associated toxicity and achieve significant cost savings for the National Health Service (NHS).

**Methods:**

We are recruiting patients with fully resected high risk stage II and stage III CRC who are being considered for ACT into the study which uses results from a plasma-only ctDNA assay to guide treatment decisions. Eligible patients are randomised 1:1 to receive ctDNA-guided chemotherapy versus SOC chemotherapy. The primary endpoint is the difference in DFS at 3 years between the trial arms. Secondary endpoints include the proportion of patients in the ctDNA-guided arm who are ctDNA negative post-operatively and receive de-escalated ACT compared to the standard arm, the difference in overall survival (OS), neurotoxicity and quality of life between the arms, and the cost-effectiveness of ctDNA-guided therapy compared to SOC treatment. We hypothesise that using a ctDNA-guided approach to ACT decisions is non-inferior to SOC. Target accrual is 1621 patients over 4 years, which will provide a power of 80% with an alpha of 0.1 to demonstrate non-inferiority with a margin of 1.25 in survival of the ctDNA-guided approach compared to SOC. We anticipate approximately 50 UK centres will participate. The study opened with the Guardant Reveal plasma-only ctDNA assay in August 2022.

**Discussion:**

The trial will determine whether ctDNA guided ACT is non-inferior to SOC ACT in patients with fully resected high risk stage II and stage III resected CRC, with the potential to significantly reduce unnecessary ACT and the toxicity associated with it.

**Trial registration:**

NCT04050345.

## Introduction

Colorectal cancer (CRC) is the fourth most prevalent cancer in the UK accounting for more than 11% of all new cancer diagnoses [1], with 42,900 patients being diagnosed every year [2]. More than 60% of these patients have early stage disease (stage I, II or III) which is potentially curable [3]. The mainstay of treatment for patients with early colon cancer is surgery, plus adjuvant chemotherapy (ACT) in those whose tumours demonstrate high risk histopathological features for recurrence [4]. Early rectal tumours may be managed with surgery alone, or with neo-adjuvant radiotherapy with or without concurrent chemotherapy, followed by surgery in those with high risk features. This may be followed by ACT if patients have not received neo-adjuvant chemotherapy in the context of total neo-adjuvant treatment (TNT) [5].

The 5 year disease free survival (DFS) for patients with stage II and III colon cancer treated with surgery without ACT is 81.4% and 49.0% respectively [6], with the risk of recurrence being highest in the first 2 years following surgery [7]. ACT aims to help reduce the risk of future recurrence by targeting potential micrometastatic disease. Current standard management of patients with resected high risk stage II and stage III CRC is 3 to 6 months of fluoropyrimidine (FP)-based ACT. ACT treatment decisions are made based on the stage and histopathological features of the resected tumour together with consideration of the patient’s clinical situation including performance status (PS). Accordingly, patients may receive 3 to 6 months of FP chemotherapy in combination with oxaliplatin; oral capecitabine plus oxaliplatin (CAPOX) every 3 weeks for 4 cycles or infusional 5-fluorauracil plus oxaliplatin (FOLFOX) every 2 weeks for 12 cycles. Alternatively, those with a poorer PS and/or lower risk histopathological features may receive 6 months of FP monotherapy, either capecitabine or 5-fluorauracil.

In stage III disease, treatment with a FP reduces the risk of cancer recurrence by 41% [8], with a further 6.9% absolute improvement in 3 year DFS from the addition of oxaliplatin [9]. Oxaliplatin-based treatment comes at a cost, however. In one study of 346 patients, 89% of patients experienced at least one symptom of acute neuropathy (e.g., sensitivity to the cold) with the first cycle of oxaliplatin. After 18 months, only 19% patients reported more than 30% reduction in symptoms according to the Chemotherapy-Induced Peripheral Neuropathy (EORTC QLQ-CIPN20) sensory scale [10]. In stage II disease, ACT with FP improves survival by 3.6% [11] with oxaliplatin offering a 5% absolute improvement in 3 year DFS [9].

The UK SCOT study established that reducing ACT duration with CAPOX from 6 to 3 months did not compromise 3 year DFS and reduced the toxicity burden of treatment [12]. These results were later pooled with other global randomised trials in the International Duration of Adjuvant Therapy (IDEA) collaboration which reported a 0.4% difference in 5 year OS, concluding 3 months of CAPOX was non-inferior to 6 months. As a result, 89.5% clinicians changed practice to offer 3 months of chemotherapy for some patients, continuing to preference 6 months of treatment in high risk stage III patients [13]. This is now widely considered the standard of care (SOC) ACT regimen for patients with fully resected CRC. Despite this evidence, it is likely that we are still over treating many patients with ACT, who may be cured with surgery alone.

Currently considerations regarding the need for ACT include histopathological features of the resected tumour and other patient factors such as age, comorbidities, PS and patient preference, leading to shared decision making. ctDNA has emerged as a biomarker for minimal residual disease (MRD) and can predict early relapse, offering a tailored approach to individual decision making [14]. Stage II patients who are ctDNA negative post-operatively have been shown to have a 3 year recurrence free survival (RFS) of 90% compared to 0% in those who are ctDNA positive [15]. Similarly in stage III disease 3 year RFS is 76% and 47% respectively [16]. Recent data from an observational registry, suggests that ACT in patients who are ctDNA negative makes little difference to DFS [17] and is potentially exposing patients to unnecessary toxicity which could be safely avoided. By reducing or omitting needless treatment in post-operative ctDNA negative patients, they may be spared the short and long-term side effects of treatment, multiple hospital visits, as well as the potential psychological impact of treatment. Should this study be positive and adopted into standard practice, there could be huge cost-saving implications for the National Health Service (NHS).

We hypothesise that ACT decisions guided by the post-operative ctDNA result will enable biomarker driven selection of patients who would and would not benefit from ACT, and thereby reduce the proportion of patients receiving ACT without compromising DFS. The primary objective is to demonstrate that a de-escalation strategy of ctDNA guided adjuvant chemotherapy is non-inferior to SOC chemotherapy as measured by 3 year DFS in patients with high risk stage II or stage III resected CRC with no evidence of MRD (i.e., those who are ctDNA negative post-operatively).

## Methods

### Study design

**Fig. 1:**
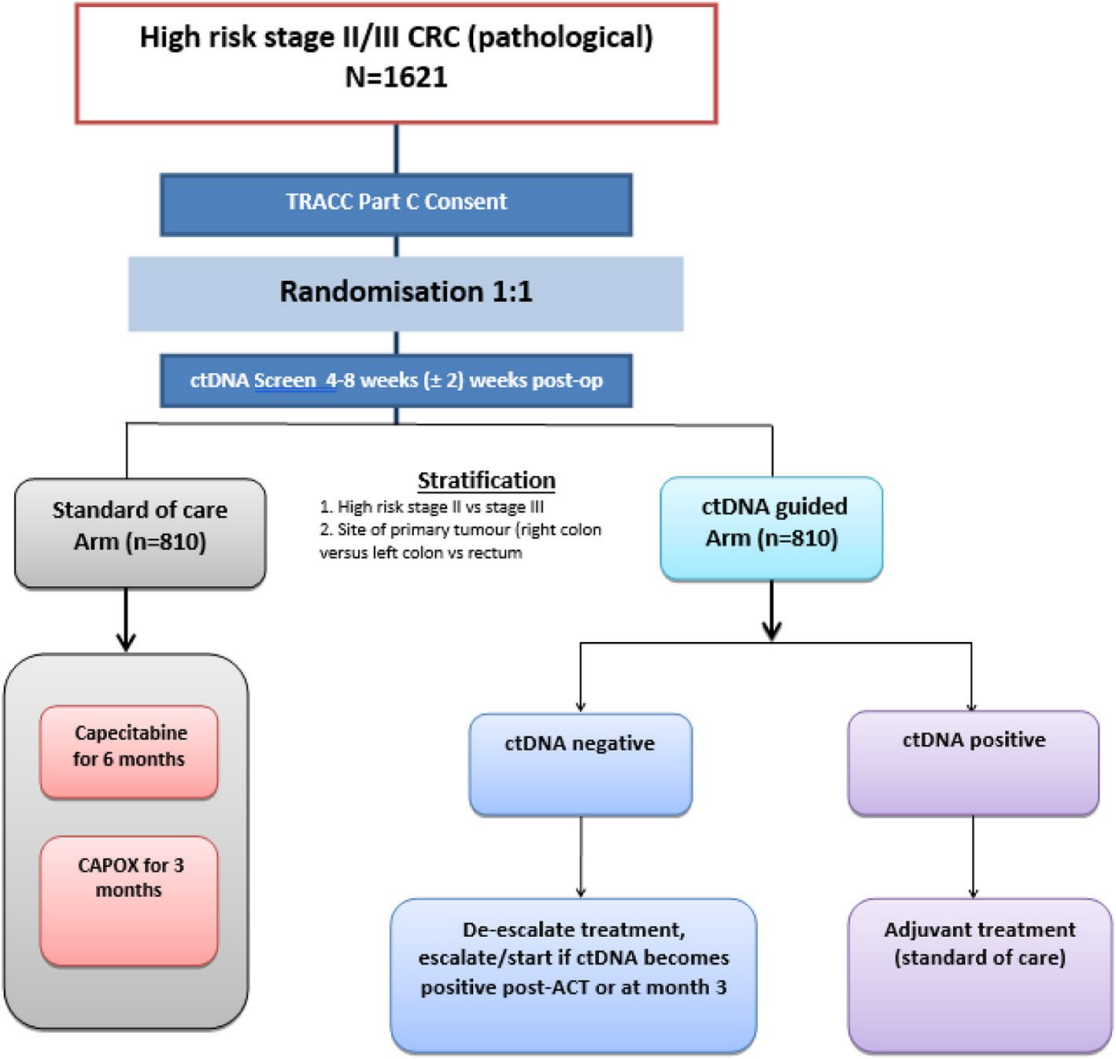
Study schema for TRACC Part C

TRACC Part C is a multi-centre, prospective, randomised study of patients with resected high risk stage II and stage III CRC who have undergone curative surgery with an R0 resection designed to demonstrate non-inferiority of ctDNA guided ACT versus SOC chemotherapy. Patients with rectal cancer who have undergone neo-adjuvant radiotherapy with or without concurrent chemotherapy (but not TNT) are also eligible. A list of participating sites is available at Tracking Mutations in Cell Free Tumour DNA to Predict Relapse in Early Colorectal Cancer—Full Text View—ClinicalTrials.gov. Eligible patients are randomly assigned to receive either ctDNA guided ACT (intervention; Arm A) or SOC chemotherapy (comparator; Arm B) (Fig. 1). Randomisation in a 1:1 ratio is performed centrally at the Institute of Cancer Research – Clinical Trials and Statistical Unit (ICR-CTSU), by random permuted blocks, with results communicated by telephone. The randomisation will be stratified by the following factors: high risk stage II versus stage III, and site of primary tumour (right colon versus left colon versus rectum).

### Study population

Patients are eligible if they are 18 years or over, have histologically proven high risk stage II or stage II CRC that has been fully resected with clear margins (> 1 mm), Eastern Cooperative Oncology Group (ECOG) PS 0–2 and able to give informed consent. Patients must have adequate organ function as determined by routine full blood count and biochemistry blood tests, and no evidence of metastatic disease on pre- or post-operative imaging, with the absence of major post-operative complications. Patients should be assessed by an oncology team and deemed suitable for treatment with ACT, having a post-operative blood sample for ctDNA collected 4–8 (+ 2) weeks after surgery in four 10.0 ml Streck cfDNA BCT® whole blood collection tubes and commencing ACT within 12 weeks. The inclusion and exclusion criteria are outlined in Table 1. Eligible patients will receive a patient information leaflet at least 24 h prior to consent and then be required to sign a written informed consent form. Consent will be taken by a clinician who is familiar with counselling patients and prescribing ACT for CRC. Successfully recruited patients who fulfil the eligibility criteria will be randomised to receive SOC ACT or ctDNA guided chemotherapy.

**Table 1:** TRACC Part C eligibility criteria

Inclusion criteria	Exclusion criteria
1. Subject ≥ 18 years of age 2. Subjects with histologically proven high-risk stage II or stage III colon or rectal cancer treated with curative intent with surgery alone (any T, N1 or N2) with no evidence of metastatic disease. High-risk stage II is defined as having one or more of the following: T4 disease, tumour obstruction and/or perforation of the primary tumour during the pre-operative period, inadequate nodal harvest as indicated by < 12 nodes examined, poorly differentiated grade on histology, perineural invasion, peritoneal involvement or extramural venous/lymphatic invasion. Subjects must be due to receive adjuvant chemotherapy following surgery Subjects with histologically proven locally advanced rectal cancer treated with neoadjuvant chemoradiotherapy (any T, N1 or N2, M0) with no evidence of metastatic disease are eligible. Subjects must be due to receive adjuvant chemotherapy following surgery 3. Fully surgically resected tumour (R0) with clear resection margins (i.e., > 1 mm) 4. Adequate organ function - Absolute neutrophil function ≥ 1.0 × 10^9^/ L - Platelet Count ≥ 75 × 10^9^ / L - Haemoglobin ≥ 80 g/L (blood transfusion before randomisation is allowed) - Adequate renal function as calculated by Cockcroft and Gault equation (GFR ≥ 50 ml/min if single agent capecitabine or CAPOX being administered) - Aspartate aminotransferase/ Alanine aminotransferase levels ≤ 2.5 upper limit of normal 5. Absence of major post-operative complications or other clinical conditions that, in the opinion of the investigator, would not contraindicate adjuvant chemotherapy 6. Patients should be assessed by Oncology team for suitability and assessment for adjuvant chemotherapy, be able to have post-operative ctDNA sample collected and be randomised by week 4–8 (± 2 weeks) after surgery and commence adjuvant chemotherapy within 12 weeks after surgery 7. ECOG performance status 0- 2 8. Able to give informed consent	1. History of concurrent and previous malignancy within the last 5 years, with the exception of non- melanomatous skin cancer and carcinoma in situ 2. Any major post-operative complications or other clinical conditions that in the opinion of the investigator would contra-indicate adjuvant chemotherapy 3. Any subject not due to receive adjuvant chemotherapy will not be eligible for Part C of the study 4. Hypersensitivity or contraindication to the drug(s) associated with the planned choice of systemic chemotherapy (CAPOX or capecitabine) as stated in the Summary of Product Characteristics (SmPC) for each of the drugs 5. Subjects due to receive 5-flurouracil (5FU) based adjuvant chemotherapy (either single agent 5FU or in combination with oxaliplatin) will not be eligible for Part C of the study, these patients will continue to be followed in the observational Part B of the study

ECOG performance status, Eastern Cooperative Oncology Group performance status

### Treatment

All chemotherapeutic agents are SOC treatments and will be administered as per local policy in terms of patient assessment, chemotherapy dose and frequency. Dose reductions due to toxicity or dihydropyrimidine dehydrogenase (DPYD) mutations will take place according to local hospital guidelines. The use of raltirexed will be accepted in the context of cardiotoxicity.

#### Arm A: SOC ACT

Patients randomised to SOC chemotherapy will be recommended 3 months of CAPOX or 6 months of capecitabine monotherapy by their clinician depending on histopathological features of their resected tumour and clinical situation. Post-operative (Month 0) and serial blood samples for ctDNA will be biobanked and processed at a future date. Patients in the SOC arm will not receive ctDNA results in real time.

#### Arm B: ctDNA guided ACT

**Fig. 2:**
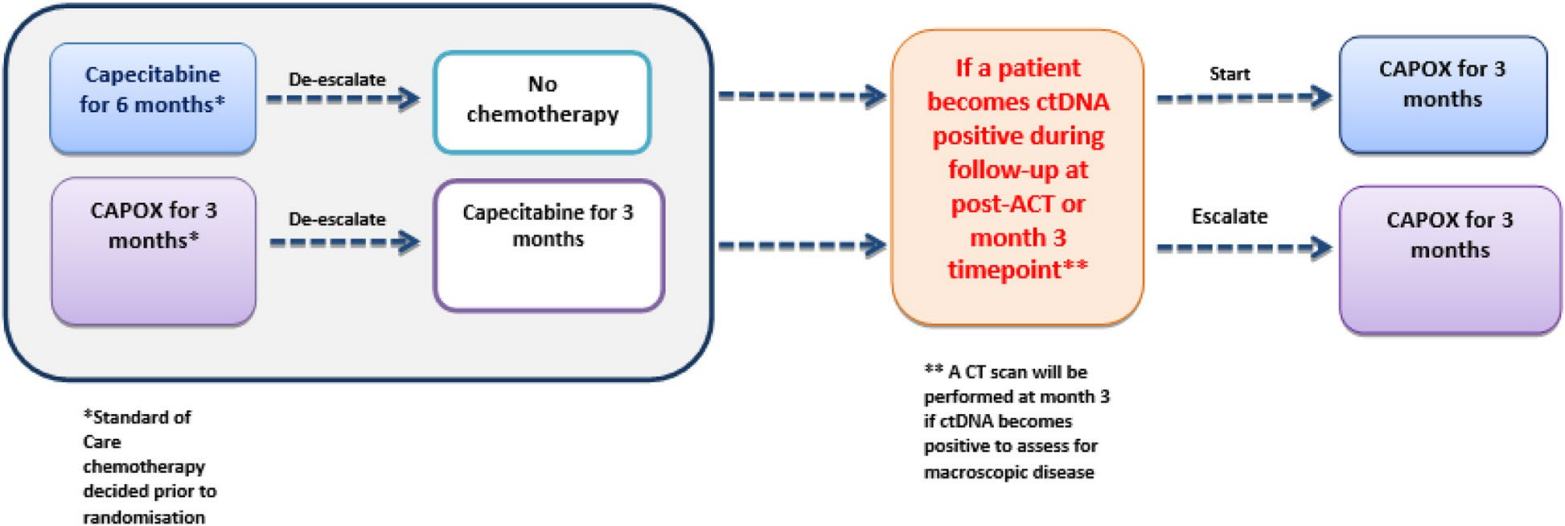
De-escalation/escalation strategy in ctDNA negative group in the ctDNA-guided arm

For patients randomised to ctDNA guided ACT, the post-operative (Month 0) blood sample will be processed in real time and the result will be published on the Guardant Health online portal. The recommended SOC chemotherapy regimen will continue to be used in patients who are ctDNA positive post-operatively, whilst ACT will be de-escalated in those who are ctDNA negative. Where 3 months of CAPOX has been recommended, treatment will be de-escalated to 3 months of capecitabine alone, and where 6 months of capecitabine monotherapy has been recommended, treatment will be de-escalated to no chemotherapy. Patients who are ctDNA negative post-operatively and have their chemotherapy de-escalated (Fig. 2) will undergo a further blood test for ctDNA post-ACT or 3 months after the post-operative sample in those who are not receiving ACT. Patients who remain ctDNA negative at the post-ACT (Month 3) timepoint will continue to follow up. If ctDNA becomes positive, chemotherapy will be escalated to 3 months of CAPOX in all patients. A CT scan will also be performed to rule out radiological macroscopic disease.

#### Follow up

**Fig. 3:**
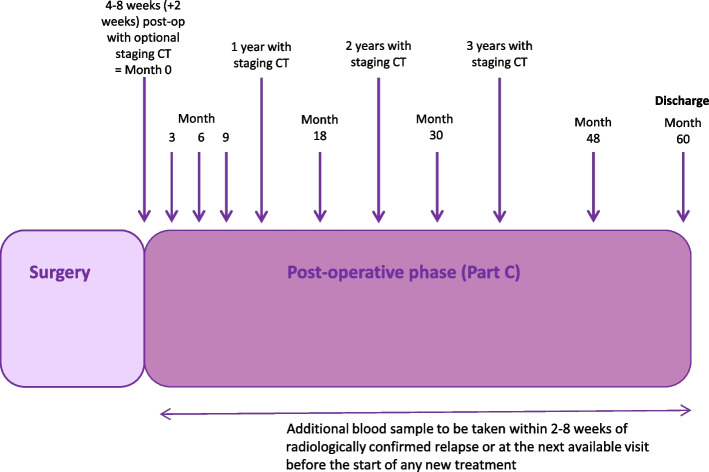
Follow up schedule for blood sample collection and CT imaging

Eligible patients will continue treatment unless there is evidence of recurrent disease, unacceptable toxicity or withdrawal of consent from the study. Following completion of adjuvant treatment, patients will undergo longitudinal blood tests for ctDNA every 3 months for the first year post-surgery, every 6 months for years 2 and 3, and annually for years 4 and 5, with computer tomography (CT) imaging performed at the end of years 1, 2 and 3 (Fig. 3). Patients will be followed up for a total of 5 years. In the instance of disease relapse, a blood sample will be taken 2–8 weeks after clinical or radiological confirmation of recurrent disease. Patients will continue follow up in the clinical trial as outlined until death, discharge from routine follow up (5 years), or withdrawal from the study. Patients whose disease recurs will be followed up for survival annually but no further blood samples will be collected.

#### ctDNA assay

In collaboration with Guardant Health, we will use the Guardant Reveal ctDNA assay to analyse blood samples. The Guardant Reveal assay is the first and only blood-based tumour-naïve ctDNA assay to detect MRD in early CRC. It leverages several technical advances in ctDNA detection to improve assay sensitivity without requiring a priori knowledge of the tumour genotype. The technique combines genomic and methylation features with independent analyses occurring in parallel to increase sensitivity. It has a turnaround time of 7–14 days for results processed in real time for clinical use. It is Clinical Laboratory Improvement Amendments (CLIA) certified and was approved by the Food and Drug Administration (FDA) in March 2021, and is already in use in three global interventional clinical trials [18–20]. Blood samples taken at the post-operative (Month 0), post-ACT (Month 3), longitudinal and relapse timepoints up to 24 months will be analysed in the first instance. Only post-operative and, if negative, post-ACT (Month 3) blood samples in patients randomised to the ctDNA-guided arm will be analysed in real time, with the remaining samples being bio-banked for future analysis.

### Quality of life and health economic analysis

Quality of life and cost-effectiveness of treatment will be assessed at each time point during the study, i.e., at baseline, post-operatively, every 3 months for year 1, every 6 months for years 2 and 3, and annually for years 4 and 5. Quality of life data related to chemotherapy-induced peripheral neuropathy will be collected using the FACT/GOG-Ntx4 subscale. Additional quality of life data will be collected using European Organisation for Research and Treatment of Cancer Quality of Life Questionnaire (EORTC QLQ-C30), the Colorectal Cancer-Specific Quality of Life Questionnaire (QLQ-CR29) and the EuroQoL 5-Dimension 5-Level (EQ-5D-3L) subscale. A pilot will be undertaken on 40 patients to assess the frequency with which questionnaires are successfully completed, with a view to adapt the frequency and length of the RUtINE™ questionnaire should the response rate be low.

### Study endpoints

#### Primary endpoint

The primary endpoint is the difference in DFS at 3 years between the ctDNA-guided arm and the SOC arm. DFS is measured from the time of surgery to recurrence, death from any cause or censored from the last follow up. Recurrence will be based on investigator assessment of clinical or radiological evidence of disease relapse. The analysis population will include all patients treated on the study. The primary population for analysis will be the Intent to Treat (ITT) population, defined as all patients randomised to treatment arms; SOC chemotherapy or ctDNA guided ACT. A sensitivity per protocol analysis will also be performed defined as all those receiving treatment as planned per randomisation.

#### Secondary endpoints

- The proportion of patients in the ctDNA guided arm receiving SOC ACT
- The proportion of patients in the ctDNA guided arm who are ctDNA negative post-operatively who become ctDNA positive during follow up and receive chemotherapy as an escalation of treatment
- The difference in overall survival (OS) between the two arms, measured from the time of randomisation to death from any cause
- The difference in neurotoxicity between the two arms, with data based on FACT/GOG-Ntx4 and common terminology criteria of adverse events (CTCAE) version 5
- The difference in quality of life between the two arms, with data based on EORTC QLQ-C30 and CR29 and EQ-5D-3L
- The cost-effectiveness of ctDNA guided arm compared to the SOC arm, with data based on a dedicated health economic questionnaire (RUtINE.™)

### Data collection and management

The Royal Marsden MACRO database will be used for clinical data collection and recording of anonymised patients and central management of the data. This may be initially via case report forms (CRFs). All staff will be trained to use the software appropriately prior to involvement in the study. As far as possible, any missing or incongruous data fields will be chased with sites for data input or clarification.

### Statistical analysis and sample size

The 3 year DFS in the SOC arm is expected to be 75%. To demonstrate non-inferiority in survival with a power of 80%, alpha of 10% (2-sided), and non-inferiority hazard ratio of 1.25 (ruling out 69.8% 3-year DFS), a sample size of 810 patients in each arm is estimated with a total of 530 events required for the analysis. As per the statistical design, 1621 patient are required to be recruited and randomised (approximately 810 per arm). Accrual will take place over 4 years across approximately 50 UK sites. An Independent Data Monitoring Committee (IDMC) will meet regularly to review the data, and particularly with regards to safety and futility. Any decision by the IDMC to discontinue the trial due to lack of efficacy will endorsed by the Trial Steering Committee (TSC).

### Planned recruitment

With a target recruitment of 1621 patients and recruitment ongoing for 4 years, at least 34 patients will need to be recruited per month. We plan to activate up to 50 UK sites, many of which are already participating in our TRACC Part B observational study. Therefore, at least 1–2 patients would need to be recruited at each site per month on average, once all sites are open. We anticipate this recruitment target is achievable given the prevalence of this tumour type and stage in the UK.

## Discussion

We describe the protocol of the multi-centre, prospective, randomised trial designed to compare the 3 year DFS in patients with high risk stage II and stage III fully resected colon and rectal cancers treated with ctDNA guided ACT versus SOC chemotherapy, sponsored by the Royal Marsden Hospital NHS Foundation Trust, London. The study builds on the results from TRACC Part B; the observational, prospective, translational research study involving serial blood sampling for ctDNA pre- and post-operatively, which demonstrated an improved 12 and 24 month RFS in patients who were ctDNA negative post-operatively.

TRACC Part C is the only ctDNA-guided study of ACT in CRC running in the UK and addresses a research question with significant health economic importance. The DYNAMIC II study is the only reported study in this space to date globally, which provided evidence to support the de-escalation of ACT in patients with resected stage II colon cancer [21]. We anticipate that the results of this study, together with the DYNAMIC II study and similar ongoing studies across the world will generate the evidence to support the clinical utility of ctDNA in the MRD de-escalation setting, generating support for a change to standard practice. In doing so, it may spare patients unnecessary chemotherapy and its associated toxicities and saeg the health service significant costs, redirecting resources elsewhere. The use of chemotherapy in this setting may halve.

TRACC Part C is one of the only ctDNA-guided studies using a plasma-only tumour-naïve ctDNA assay for MRD detection in CRC globally. Utilising a blood-only assay with a turnaround time of 7-14 days, where analysis of tumour tissue is not required, lends itself to a more streamlined approach to recruitment within the study, as well as smooth potential future implication into standard clinical practice. The inbuilt patient reported outcomes (PROs), health economic analysis and process evaluation will provide a route to effective implementation in SOC clinical practice within the NHS and elsewhere in the future.

## Data Availability

Data sharing not applicable as no datasets generated and/or analysed for this study to date.

## Abbreviations

ACT: Adjuvant chemotherapy
CAPOX: Capecitabine and oxaliplatin
CEA: Carcinoembryonic antigen
CI: Confidence interval
CRC: Colorectal cancer
CRF: Case report form
CT: Computer tomography
ctDNA: Circulating tumour DNA
DFS: Disease free survival
DPYD: Dihydropyrimidine dehydrogenase
EORTC: European organisation for research and treatment of cancer
FOLFOX: 5-Fluorouracil and oxaliplatin
FP: Fluoropyrimidine
IDMC: Independent Data Monitoring Committee
MRD: Minimal residual disease
NHS: National health service
OS: Overall survival
PS: Performance status
REC: Research ethics committee
RFS: Recurrence free survival
SOC: Standard of care
TNT: Total neo-adjuvant treatment
TSC: Trial Steering Committee
